# Peripheral blood lymphocytes are able to maintain their viability and basic function in normal urine

**Published:** 2016

**Authors:** Azin Aghamajidi, Hesam Babaie, Narges Amirjamshidi, Zeinab Abedian, Hamidreza Khorasani, Amrollah Mostafazadeh

**Affiliations:** 1 Department of Immunology**, **Babol University of Medical Sciences, Babol, Iran.; 2 Cellular and Molecular Biology Research Center, Babol University of Medical Science, Babol, Iran.

**Keywords:** Urine, lymphocyte lifespan, lymphocyte activation, PHA

## Abstract

**Background:**

Similar to inflammatory cells, peripheral blood mononuclear cells (PBMCs) can also infiltrate in to kidney and urinary tracts and subsequently excreted by urine. In this study we determined the viability rate and response to phytohemagglutinin-A (PHA) of human PBMCs in normal urine.

**Methods:**

A number of 1×10^6^ ficoll-hypaque isolated PBMCs were dispensed in 1 ml normal urine and 6 molar urea and RPMI-1640+FBS10 % were considered as negative and positive control, respectively. After 20, 60 and 120 minutes the viability of these cells was measured by trypan blue dye exclusion assay. 1×10^5^ of PBMCs were isolated from urine and cultured as triplicate in RPMI-1640`supplemented with FBS 10% and PHA for 96hr. MTT assay was performed to determine the PBMCs response to PHA. These experiments were repeated three times independently.

**Results:**

There was no significant difference between the viability rates of the PBMCs incubated in urine and positive control after 20, 60 and 120 minutes. Overall, there was a significant difference in trends of viability rate across the three groups (p<0.05).

**Conclusion:**

Our results showed that not only PBMCs remained remarkably alive in urine after 120 minutes, but can also respond to PHA up to 60 minutes after incubation in urine. These data open a new avenue in the designation for cell culture-based techniques in urine cell analysis.

Similar to inflammatory cells, lymphocytes and monocytes infiltrate in any tissue including the kidneys, the upper and lower urinary tract systems, and subsequently are excreted in the urine (1). Normal urine is yellowish clear fluid, with PH of 4.5-8, gravity range of 1.003-1.035 and osmolality of 500-800 mOsmol/Kg which has no glucose, protein and microorganism (2). Leukocytes enter it urine in different pathological conditions such as prostatic, bladder and kidney cancers, urinary tract, prostate and kidney infections and inflammations or in some autoimmune disease like lupus nephritis (3). Urine mononuclear cells can be divided into different groups based on the location of their entrance to the urine. In some diseases like lupus glomerulonephritis, these cells can be originated directly from peripheral blood and in other conditions they can enter to urine from inflamed urethra without storage in bladder. These leukocytes expected to carry some important information from scene of inflammatory reactions develop in involved tissues and organs. Inflammatory/anti-inflammatory cytokine and chemokine patterns at a single cell level of urine derived mononuclear cells by some technique such as Elispot, are useful tumor/inflammatory markers for a variety of urinary system cancers and inflammatory conditions.

Thus, urine derived mononuclear cells may be a good alternative to replace some invasive diagnostic methods like kidney biopsy for diagnosis or monitoring treatment of such conditions. To perform such techniques, we need to culture the urine derived mononuclear cells at least for three days in standard cell culture condition. However, the main concern is that leukocytes cannot be preserved alive or keep their activity at an optimal level in urine for a polong time since isolation from inflicted tissues to urine to be stored in the bladder for average period of 5 h, is due to the presence of harsh conditions in urine such as hyper osmolality. Therefore, knowledge about the dynamic PBMCs (peripheral blood mononuclear cells) resistance to urine somehow to maintain both their viability and function at acceptable levels needs to establish some new reliable research and diagnostic methods based on urine derived cells analysis. 

Accordingly, we determined not only the viability rate of human PBMC dispensed in fresh normal urine, but also stimulated the urine treated PBMC with PHA (Phytohaemagglutinin A) for four days in standard cell culture condition to evaluate the lymphocyte proliferation rate as a basic activity of immune cells that occurs in response to antigens.

## Methods

**Viability assessment: **6 ml of blood is taken from a healthy donor through venipuncture (female, age: 24) at three different occasions. 1.2×10^7^ PBMC were isolated from blood by ficoll-hypaque gradient centrifugation (1314g, 30 min) method. The isolated PBMCs were dispensed in 12-well cell culture plate (10^6 ^cells per well) with 1ml fresh normal urine which was prepared from a 27-year old man and incubated at 37°c, 5% CO_2_ for three different time points (20, 60 and 120 min). The same number of PBMCs were incubated in 1 ml of 6 M urea in 0.2 M phosphate buffered saline (PBS) and RPMI-1640+ FBS 10 % (ATOCEL, Austria) as negative and positive control, respectively, for each time point. After indicating time the viability of the cells was measured with trypan blue dye exclusion assay and cell count was obtained by Neubauer slide. This experiment was repeated three times independently.

**MTT assay: **MTT assay was conducted to assess urine treated lymphocyte function (according to MTT Cell Proliferation Assay, ATCC® 30-1010K). 1×10^5 ^of PBMCs which had been already incubated in urine, were isolated from urine by micro centrifuge (EDISON, NJ USA) and then the cells were stimulated by PHA 1.5% (Sigma-Aldrich, Germany) in RPMI-1640 medium supplemented by FBS 10% as triplicate under standard cell culture condition in 96 –well cell culture plate. After 96 h, the necessary microphotographies were prepared by inverted microscope equipped with Olympus (U-TV0.63XC, Japan) and then 50µl (5mg/ml) of yellow tetrazolium MTT (3-(4, 5-dimethylthiazolyl-2)-2, 5-diphenyltetrazolium bromide) (Sigma-Aldrich, Germany) was added to each well and incubation was continued for another 4 h. Then acidic isopropanol was added and mixed to solve formazan crystals and the absorbance of developed colour (OD) was determined in 570 and 630 nm with ELISA reader (Ray2 China). This experiment was also repeated three times independently.

**Statistical analysis: **Microsoft office (Excel 2013) was used for data analysis. T-test was used to compare the mean values of viability. P<0.05 was considered a significant level. 

## Results

**Figure 1:**
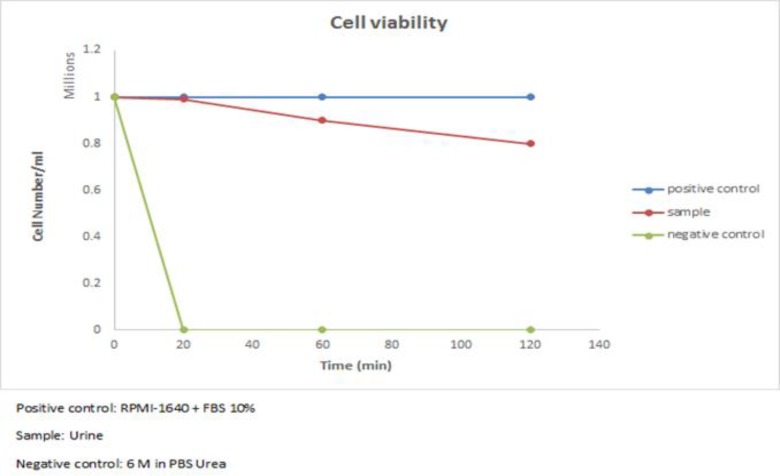
Trypan blue staining result -Viability rate of lymphocytes incubated in urine for 3 different time points (20.60.120 minutes). Lymphocytes incubated in urine survive for 20 min but 20 percent of them die after 2h but this decrement was not statistically significant in comparison with positive control (lymphocytes incubated for the same times in RPMI-1640 + 10% FBS). 6M urea incubated lymphocyte died rapidly during the first 20 min of incubation

**Figure 2:**
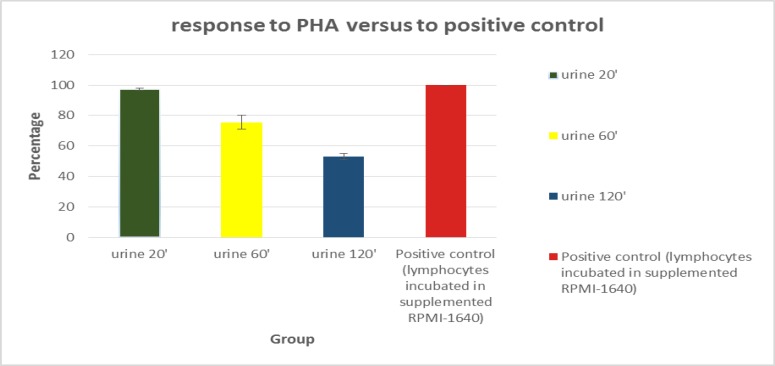
Lymphocyte’s response to PHA and their function compared to control group; the activity of lymphocytes incubated in urine for 20 min is 97% of control group. It shows that the proliferation rate of these lymphocytes is similar to lymphocytes incubated in supplemented RPMI-1640. Lymphocytes incubated in urine for 120 min keep almost 50% of positive control activity

We designed an in-vitro model to estimate the in-vivo half-life of lymphocyte in urine which they can enter into it from a long route from kidney to bladder and finally excreted by urine. To this end, the PBMCs were dispensed in urine and incubated for different time points. Then the viability and function of these cells were determined. As figure1 indicated, we were not able to find any significant difference in the viability rate of lymphocytes incubated in urine for 20, 60, 120 min and lymphocytes which were cultured for the same time in a standard cell culture condition; but as it could be expected there was a significant difference between the trend of viability rate of lymphocyte incubated in 6M urea in PBS and lymphocytes cultured in RPMI-1640/FBS 10% or lymphocytes incubated in urine (P<0.05). Interestingly, there was no significant difference in response to PHA between lymphocyte incubated for 20 min in urine and lymphocyte cultured in RPMI-1640 for the same time (fig 2). 

**Figure 3:**
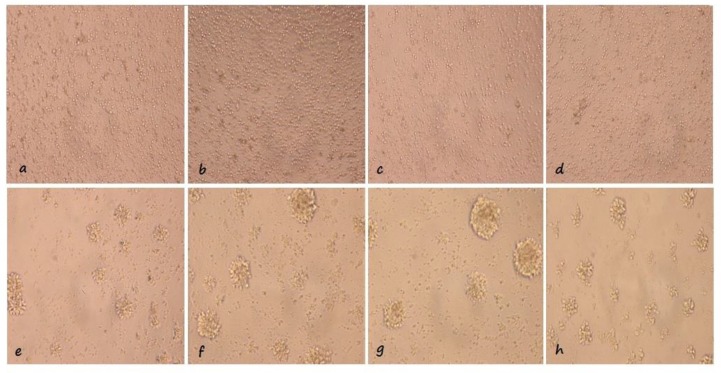
Morphology analysis of lymphocyte’s response to PHA; Lymphocytes incubated in urine for (a) 20, (b) 60 and (c) 120 min, non-stimulated with PHA; (d) Lymphocytes which dispensed in RPMI-1640 as a positive control, non-stimulated with PHA. Lymphocytes incubated in urine for (e) 20, (f) 60 and (g) 120 min, stimulated with PHA; (h) Lymphocytes which dispensed in RPMI-1640 as a positive control stimulated with PHA; Lymphoblasts in part e and h are similar in size and number. These microphotographies showed agglutination of lymphocytes in response to T cell stimulator

However, after 60 min this response reached to 71% of its positive control response namely lymphocytes incubated in RPMI-1640 for 60 min and stimulated with PHA for 96 h and after 120 min the response diminished to only 53% of positive control. Our microphotography and the data originated from viability/function assays were consistent. Pre-incubated lymphocytes for 20 min in urine, were round and shiny (3a). These cells stimulated with PHA formed blast cells (3e) similar to the control cells (3h). The lymphocytes which were pre incubated in urine for 60 min also formed blast cells when stimulated with PHA for four days (3f) in comparison to non-stimulated lymphocytes (3b) surprisingly, 120 min urine pre- incubated lymphocytes transformed to blast cell (3g) but the MTT assay data showed a decrement in their activity (fig 3).

## Discussion

In some chronic inflammatory conditions such as viral infections, bladder stones, renal transplant rejection, tumors, prostatitis and urethritis lymphocytes can be seen in the urine. The existence of lymphocytes in urine represents inflammatiory process in the organs and can be used as a diagnostic tool for such disease but the half-life of these cells in the urine is unclear. This study was designed to obtain a precise estimation about lymphocyte lifespan and their function dynamic in urine. The remarkable finding of our study was that the lymphocytes could be alive in urine for 2 h while keep their activity for 60 min at a significant level. Jacobson reported that about 80-90 percent of urine derived lymphocytes remained alive for 72 h in cell medium-urine mixture in the presence of PHA, concavalin A and pokeweed mitogen. In contrast to our study which blood isolated lymphocytes were dispensed directly in the first morning random normal urine, they mixed urine with supplemented cell culture medium as 1:1(4). Moreover, Jo´zef Stachowski’s studies revealed that in patients who received renal transplants, 72-90 % of urine derived lymphocytes remained alive 2 h after urination, in urine with osmolality of 300-700 mosmol. If we increase urine retention time in bladder (5 h averagely) to 2 h, we reach to 7 h as a lifespan for more than 2.3 of lymphocyte in urine. We did not investigate the lifespan of lymphocyte beyond 2 h but the trend of the curve shows that certainly it is less than 7 h (fig 1). We suppose that this difference is due to different levels of protein existing in urine of healthy individual as compared to a transplanted one. Proteinuria is a common phenomenon in individuals with kidney transplantation (5-7). Some basic function of urine pre incubated lymphocytes such as proliferation was examined to evaluate the urine derived lymphocyte application in some cell culture based techniques like elispot or intracytoplasmic flowcytometry. We found that after 60 min, more than 2/3 and after 2 h 50% lymphocyte can keep their function in urine. Thus, we can use lymphocytes which enter to urine from lower urinary tract, prostate and maybe bladder and glomerule in different pathological conditions like cancer or sterile inflammatory diseases such as lupus glomerulonephritis for cell culture-based techniques of urine analysis. If we assume that in pathological condition like glomerulonepheritis or in kidney rejection process, the protein levels increase in urine and so according to Jo’zef Stachowski’s study, the existence of protein increases the urine lymphocyte lifespan, this conclusion appears to be reasonable. 

Further studies are needed to evaluate the stability of protein, DNA and RNA especially micro RNA of these cells in urine. Taken together, the data in this study showed that lymphocytes are able to maintain their viability and their basic function in harsh condition like urine. These data open a new avenue for cell culture-based techniques in urine cell analysis.

## Notes

**Funding:** This work was financially supported by Cellular and Molecular Cell Biology Research Center at Babol University of Medical Sciences.

## References

[1] Ringsrud KM (2001). Cells in the urine sediment. Laboratorymedicine.

[2] Ben-Ezra J, Zhao SH, McPherson RA, McPherson RA, Pincus MR, Henry JB (2007). Basic examination of urine. Henry's clinical diagnosis and management by laboratory methods.

[3] De Boer EC, De Jong WH, Van Der Meijden AP (1991). Presence of activated lymphocytes in the urine of patients with superficial bladder cancer after intravesical immunotherapy with bacillus Calmette-Guerin. Cancer Immunol Immunother.

[4] Corriere JN Jr, Jacobson KA (1976). The effects of urine on the viability and activity of lymphocytes. J Urol.

[5] Stachowski J, Barth C, Lewandowska-Stachowiak M (1998). Flow cytometric analysis of urine lymphocytes isolated from patients with renal transplants--purification of urine lymphocytes. J Immunol Methods.

[6] Amer H, Lieske JC, Rule AD (2013). Urine high and low molecular weight proteins one-year post-kidney transplant: relationship to histology and graft survival. Am J Transplant.

[7] Amer H, Cosio FG (2009). Significance and management of proteinuria in kidney transplant recipients. J Am Soc Nephrol.

